# Insight to Improve α-L-Arabinofuranosidase Productivity in *Pichia pastoris* and Its Application on Corn Stover Degradation

**DOI:** 10.3389/fmicb.2018.03016

**Published:** 2018-12-14

**Authors:** Fengzhen Zheng, Junquan Liu, Abdul Basit, Ting Miao, Wei Jiang

**Affiliations:** Beijing Advanced Innovation Center for Food Nutrition and Human Health, State Key Laboratory of Agro-Biotechnology, College of Biological Sciences, China Agricultural University, Beijing, China

**Keywords:** α-L-arabinofuranosidase, *Pichia pastoris*, propeptide, codon optimization, saccharification

## Abstract

α-L-arabinofuranosidase (ARA) with enhanced specific activity and in large amounts, is needed for a variety of industrial applications. To improve ARA production with engineered methylotrophic yeast *Pichia pastoris*, a genetically modified *ara* gene from *Aspergillus niger* ND-1 was investigated. Through codon optimization and rational replacement of α-factor signal peptide with the native propeptide (MFSRRNLVALGLAATVSA), ARA production was improved from 2.61 ± 0.13 U/mL to 14.37 ± 0.22 U/mL in shaking flask culture (a 5.5-fold increase). Results of N-terminal sequencing showed that secreted active ARA of recombinant strain p-oARA had theoretical initial five amino acids (GPCDI) comparable to the mature sequences of α-oARA (EAEAG) and αp-oARA (NLVAL). The kinetic values have been determined for ARA of recombinant strain p-oARA (*V*_max_ = 747.55 μmol/min/mg, *K*_m_ = 5.36 mmol/L), optimal activity temperature 60°C and optimal pH 4.0. Scaling up of ARA production by p-oARA in a 7.5-L fermentor resulted in remarkably high extracellular ARA specific activity (479.50 ± 12.83 U/mg) at 168 h, and maximal production rate 164.47 ± 4.40 U/mL. In studies of corn stover degradation activity, degree of synergism for ARA and xylanase was 32.4% and enzymatic hydrolysis yield for ARA + xylanase addition was 15.9% higher than that of commercial cellulase, indicating significant potential of ARA for catalytic conversion of corn stover to fermentable sugars for biofuel production.

## Introduction

Second-generation ethanol production is based on plant biomass degradation. The primary type of plant biomass is cellulose, hemicellulose and lignin (Saha, 2000). The predominant method for biomass degradation is polysaccharide hydrolysis by CAZymes. The CAZy database uses sequence-based means to classify enzyme families^1^. Hemicellulose (complex polysaccharide) can be degraded by various combinations of hemicellulases (Gao et al., 2011; Ravalason et al., 2012). The enzymes ARAs or arabinases degrade the complex polysaccharides or arabino-oligosaccharides and liberate L-arabinose (McKee et al., 2012). ARAs cleave α-(1,2), α-(1,3), or α-(1,5) linked L-arabinofuranosyl residues from polysaccharides or oligosaccharides containing arabinose (Bouraoui et al., 2016). In the CAZy database, ARAs are categorized into the different families of GH, GH2, GH3, GH43, GH51, GH54, and GH62 (Wilkens et al., 2017). GH54 contains several ARAs reported to act synergistically with various cell wall-degrading enzymes (Lagaert et al., 2014).

Many ARAs have potential use as tools in biotechnological and industrial applications (Numan and Bhosle, 2006). After cellulose, the hemicelluloses are considered the second most abundant biomass polymer which comprised on 20–35% of total lignocellulosic biomass (de Souza et al., 2011). Enzymatic hydrolysis of hemicelluloses is inhibited due to the presence of L-arabinosyl residues (acting as a side chain) in carbohydrate polymers (Öhgren et al., 2007). Under optimal industrial conditions, hydrolysis of complex polysaccharides into fermentable sugars is based on synergized actions of ARAs with other hemicellulose hydrolysis enzymes for degradation of plant biomass (Raweesri et al., 2008). Use of ARAs for specific or partial embellishment is a promising approach for the utilization of the economically cheap natural materials (Numan and Bhosle, 2006). Various industrial practices require sufficient amounts of ARAs with improved specific activity.

Yeasts are highly suitable hosts for expression of heterologous proteins (Šiekštelė et al., 2015). *Pichia pastoris* is a well-established and widely used protein expression system for the yield of high titer of recombinant proteins and is able to secrete proteins into media (Looser et al., 2015). Important advantages of *P. pastoris* over other protein expression hosts are its capacity to perform eukaryotic PTMs such as glycosylation, and function as a strong regulatory alcohol oxidase promoter (AOX1), which is involved in methanol utilization pathways that generate high levels of heterologous recombinant proteins (Gellissen, 2000; Basit et al., 2018b).

The fungal genus *Aspergillus* (phylum Ascomycota) is a crucial source of CAZymes with the capability of high protein secretion (Benoit et al., 2015). ARAs from different *Aspergillus* species belongs to various GH families have been identified in secretome analysis. Enzymes from *Aspergillus* species are capable of catalyzing protein N-glycosylation (an important PTM) and display desirable enzyme properties such as high stability and activity (Shirke et al., 2017). Here, we described (i) production of a novel recombinant ARA from *Aspergillus niger* strain ND-1 using *P. pastoris* expression system, (ii) target *ara* gene transcription level (qPCR) and site-specific cleavage of signal peptide from preprotein of ARA variants, and (iii) its enzyme properties, including saccharification efficiency in combination with commercial ACR and endo-1,4-β-xylanase (XYL). High-yield secretory production of stable ARA in *P. pastoris* was firstly achieved through optimization of *ara* gene codons and engineering of the N-terminal propeptide.

## Materials and Methods

### Strains, Vectors, and Reagents

*A. niger* ND-1 (GenBank Accession number MH137707) was selected from samples collected in Chifeng, Inner Mongolia, China, and maintained in our laboratory. *E. coli* DH5α, *P. pastoris* strain X-33 and plasmid pPICZαA were purchased from Invitrogen (Beijing, China). All-in-One First-Strand Synthesis MasterMix with *DNa*se I was from NOVA Yugong Biolabs (Jiangsu, China). pMD19-T sample vector, restriction enzymes, and ligases were from Takara. P-nitrophenyl (pNP) -β-D-xylopyranoside, p-nitrophenyl α-L-arabinofuranoside (pNP-α-L-Araf), pNPG, pNPC, CMC-Na, RBB-xylan, commercial endo-1,4-β-xylanase (XYL) from *Thermomyces lanuginosus* and ACR from *Trichoderma reesei* (ATCC 26921) were purchased from Sigma-Aldrich (United States). The extraction of total genomic DNA was performed by using DNeasy Blood & Tissue Kit (QIAGEN, Germany) as per manufacturer’s protocol. All chemicals were of analytical grade and commercially available.

### Sequence Analyses

BLAST program was used for the alignment of protein and nucleotide sequences of *ara*^2^ and analyzed using the DNAMAN 6.0 software program. Glycosylation sites and signal peptides of ARA were predicted by NetNGlyc 1.0 server program^3^ and SignalP 4.1 server program^4^, respectively.

### Construction of Recombinant Plasmids and Strains

Full-length genes of mature *ara* and premature *ara* (contained propeptide, *para*) were amplified from genomic DNA of *A. niger* ND-1 (Gene ID: MH023278) using primer pairs ARA-F/ ARA-R and pARA-F/ pARA-R (Supplementary Table 1). On the basis of yeast codon usage bias^5^, the *para* gene was optimized to further increase ARA expression (ZixiBio Techco., Ltd., Beijing), and the resulting gene termed *poara*.

**FIGURE 1:**
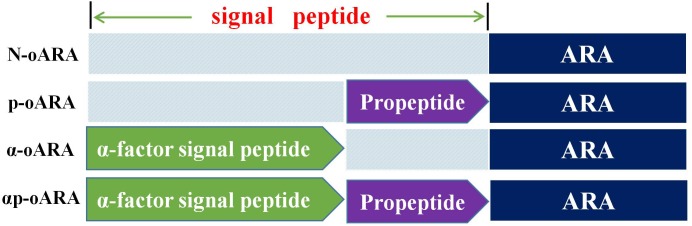
Schematic representation of the designed ARA signal peptides. The recombinant strains have different signal peptides. N-oARA (without any peptide), p-oARA (with propeptide but no α-factor signal peptide), α-oARA (with α-factor signal peptide but no propeptide) and αp-oARA (with α-factor signal peptide and propeptide).

Amplicons were purified from the gel by using Gel Extraction Kit (Thermo Fisher, United States), and then ligated to pMD19-T sample vector as per manufacturer’s protocol, transformed into DH5α, digested with *Xba*I and *Eco*RI, and connected to pPICZαA pretreated with respective enzymes. The resulting recombinant plasmids including *ara* and *poara* were termed pPICZα-*ara* and pPICZαp-*oara*. Sequenced recombinant plasmids were transformed into *P. pastoris* (X-33) by electroporation as previously described (Basit et al., 2018a), generating variants α-ARA and αp-oARA (Figure 1).

pPICZαp-*oara* encoding α-factor signal peptide and optimized premature *ara* were designed to facilitate construction of an ARA variant with propeptide or α-factor signal peptide by one-step PCR as previously described (Zhang et al., 2016). Specifically, the variant contained propeptide segment of pPICZαp-*oara* positioned between N-terminus of ARA and C-terminus of the α-factor signal peptide. pPICZp-*oara* (with propeptide sequence but no α-factor signal peptide), pPICZα-*oara* (with α-factor signal peptide but no propeptide), and pPICZ-*oara* (without any peptide) were amplified from constructed pPICZαp-*oara* plasmid with respective primer pairs (pPICZp-*oara-*F/ pPICZp-*oara-*R, pPICZα-*oara-*F/ pPICZα-*oara-*R, and pPICZ-*oara-*F/ pPICZ-*oara-*R) (Supplementary Table 1) by one-step PCR, using DNA Polymerase (Phanta Max Super-Fidelity, Vazyme Biotech Nanjing, China). Recombinant strains p-oARA, α-oARA, and N-oARA (Figure 1) were obtained by transformation of respective linear plasmids. Identification of positive transformants was based on colony PCR with primer pair (AOX-F/ AOX-R) and sequencing (Invitrogen; Shanghai, China) (Supplementary Table 1). Transformants were grown on peptone dextrose medium (YPD) agar plates (2% (w/v) glucose, 2% (w/v) tryptone (OXOID, Hampshire, England), 1% (w/v) yeast extract) containing 100 μg/mL Zeocin (Invitrogen; Carlsbad, CA, United States). Moreover, all the recombinant strains were verified for gene copy number by PCR with primer pair (pUC-F/ *ara* internal reverse primer-R) (Supplementary Table 1).

### SDS–PAGE and Amino Acid Sequencing

The concentration of protein was determined by Bradford assay using BSA as standard (Bradford, 1976). Recombinant ARAs were assayed by 12% SDS–PAGE. Protein bands of recombinant ARA were visualized by staining with Coomassie Brilliant Blue R-250 (Bio-Rad Laboratories, United States) or transferred to PVDF membrane (Bio-Rad). Edman degradation method for N-terminal amino acid sequencing was carried out using Protein Sequencer (Applied Biosystems model 491P) at Peking University (Beijing).

### Flask Culture and Fed-Batch Fermentation

Recombinant colonies of X-33 were cultured in 5 mL YPD medium for 24 h at 30°C, 200 rpm. Starter inoculum was transferred at 6% (v/v) to 50 mL buffered glycerol-complex (BMGY) medium (potassium phosphate (100 mM, pH 6.0), 1% (w/v) yeast extract, 1% (w/v) glycerol, 2% (w/v) tryptone) containing 1.34% (w/v) YNB and 4 × 10^-5^% (w/v) biotin and grown at 30°C, 200 rpm, until OD_600_ reached 5–6. Cultured cells were resuspended in 50 mL buffered methanol-complex (BMMY) medium (potassium phosphate (100 mM, pH 6.0), 1% (w/v) yeast extract, 2% (w/v) tryptone, 1% (v/v) methanol) containing 1.34% (w/v) YNB and 4 × 10^-5^% (w/v) biotin. The protein expression was induced by addition of methanol to final concentration 1% (v/v) every 12 h. The recombinant proteins were then analyzed by SDS–PAGE.

Fed-batch fermentation of ARAs was performed in a 7.5-L fermentor (Shanghai Boxing Bio-engineering Equipment Co., Ltd.) with 5 L basal salts medium (BSM) (glycerol (40 g/L), KOH (4.13 g/L), MgSO_4_⋅7H_2_O (14.9 g/L), H_3_PO_4_ (26.7 mL/L), CaSO_4_⋅7H_2_O (14.9 g/L), K_2_SO_4_ (18.2 g/L)). Inoculum (10% v/v) was obtained from YPD culture after 24 h at 30°C, 200 rpm. During initial glycerol batch phase, pH was maintained at 5.5 using 50% (v/v) ammonium hydroxide. Temperature (30°C) was maintained and DO level at > 30% by cascaded control of agitation (500–700 rpm) with aeration rate 2–10 L/min. When DO level reached > 60%, glycerol was pumped into fermentor with medium containing 50% (w/v) glycerol and 1.2% (v/v) PTM1 solution at fed-batch phase (Zhang et al., 2018). ARA was expressed via AOX1 promoter induction by methanol in methanol fed-batch phase. Residual glycerol was depleted for 2 h when DO level reached > 60%, to prevent inhibition of AOX1 promoter by glycerol (Ponte et al., 2016). Methanol containing 1.2% PTM1 was then fed by fermentor autocontrol system (linear feeding) to maintain methanol concentration ∼1% in the medium.

### Deglycosylation of Recombinant ARAs

Deglycosylation of expressed ARAs was performed by endoglycosidase-H (endo-H) enzyme (New England Biolabs). In brief, ARAs (1 mg) were mixtured with buffer (pH 4.8) followed by boiling for denaturation (10 min), treated with endo-H (0.1 U) and then incubated for 1 h at 37°C. The product was then analyzed by 12% SDS–PAGE.

### Transcription Level of Protein Expression Variants

Five variants related to target *ara* were examined. RNA extraction, cDNA synthesis, and qPCR analysis were performed as in our previous study (Huang et al., 2014). GAPDH (gene ID: 8198905; located on chromosome 2 of *P. pastoris*) was used as reference gene. Primers (F_rARA_/ R_rARA,_ F_GAPDH_/ R_GAPDH_) used for qPCR are listed in Supplementary Table 1.

### Enzyme Activity Assays

α-L-arabinofuranosidase activity was measured using soluble chromogenic substrate pNP-α-L-Araf as described previously (Bouraoui et al., 2016). The reaction mixture, containing 100 μL enzyme in sodium acetate buffer (50 mM, pH 5.0) and 100 μL pNP-α-L-Araf (5 mM), was incubated at 50°C, 10 min. The reaction was terminated using 100 μL sodium carbonate (1.0 M). Amount of pNP was evaluated based on A_405_ with reference to a standard curve. One unit (U) enzyme activity was defined as the amount of enzyme required to release 1 μmol pNP per min under our assay conditions.

Enzyme activity of commercial ACR was measured by DNS method (Miller, 1959) at 50°C in sodium acetate buffer (50 mM, pH 5.0). The activity of commercial XYL was measured using RBB-xylan as substrate (Marcolongo et al., 2014). One unit enzyme activity was defined as the amount of enzyme required to increase absorbance at 590 nm at a rate of 1 A min^-1^ under our assay conditions. All enzyme activity measurements were performed in triplicate.

### Enzyme Characterization

Effects of pH on ARA enzyme activity were determined using solutions of 50 mM (pH 2–3) glycine-HCl, (pH 3–4) sodium citrate, (pH 4–6) sodium acetate, and (pH 6–8) sodium phosphate at 50°C. pH stability was determined during 60-min periods at pH values ranging from 2 to 8, and residual activity was measured at optimal pH (4.0). Effects of temperature at optimal pH were determined based on measurements of enzyme activity (expressed as relative activity) at temperatures ranging from 20 to 90°C. Thermostability of ARA was evaluated by measuring residual enzyme activity under standard assay conditions following pre-incubation at optimal pH, and at 50, 60, or 70°C without substrate.

α-L-arabinofuranosidase activity was evaluated in the presence of 5 mM concentration of various metal ions (Ni^2+^, Co^2+^, Al^3+^, Na^+^, Mn^2+^, Zn^2+^, Ca^2+^, Cu^2+^, Fe^2+^, Mg^2+^, Fe^3+^), EDTA (5 mM), Tween-20 (0.05%), and SDS (0.1%). The blank control contained no additives.

The substrate specificity of ARA was determined at pH 4.0, 50°C with 5 mM of pNP derivatives (pNP-α-L-Araf, pNPC, pNPG, and pNPX) or 1 % (w/v) of CMC-Na and RBB-xylan as the substrate.

*V*_max_ and *K*_m_ values for ARA were measured using various concentrations (0.5 to 5.0 mM) of pNP-α-L-Araf substrate. Data were generated by Lineweaver-Burk plot method (Erithacus Software; Horley, United Kingdom). Determinations were performed in triplicate.

### Saccharification of Pretreated Corn Stover

Substrate hydrolysis has been accelerated in previous studies by a variety of pretreatment methods (biological, chemical, mechanical, thermal), singly or in combination (Lau et al., 2009; Chen et al., 2013). Corn stover (i.e., stalks, leaves, and cobs that remain in fields following corn harvest) was broken by alkaline pretreatment as previously described (Basit et al., 2018a) and then added with 5% (w/v) sodium acetate buffer (50 mM, pH 5.0). ACR and XYL were added with ARA of recombinant strain p-oARA culture supernatant in the reaction mixture and then incubated for 72 h at 50°C, 200 rpm. Samples were taken at 24-h intervals and centrifuged at 12000 rpm for 3 min. The blank controls containing substrate alone were treated under the same conditions. Amounts of reducing sugars released were determined by DNS method colorimetry with glucose as the standard. Experiments were performed in triplicate. Degree of synergism of ARA and XYL was evaluated as improving produced by the two hemicellulases separately, divided by enhance produced by the enzymes in combination, expressed as a percentage. Degradation rate (percentage of total corn stover) was calculated using corresponding formula as previously described (Basit et al., 2018a).

## Results

### Sequence Analyses

Our sequence analysis indicates that *ara* gene (Gene ID: MH023278) from *A. niger* ND-1 (GenBank Accession number MH137707) has length 1500 bp. Information available on the NCBI and CAZy database indicates that the characterized ARA belongs to GH54. The protein encoded has 499 amino acids and predicted mass 52,558 Da. The propeptide signal of *ara* gene secreted 18 amino acids (MFSRRNLVALGLAATVSA) predicted based on SignalP program. The *ara* gene was optimized by upgrading the codon adaptation index from 0.6 to 0.86 and GC content was reduced from 58.3 to 41.2%.

### Optimization and Expression of *ara* Codons

**FIGURE 2:**
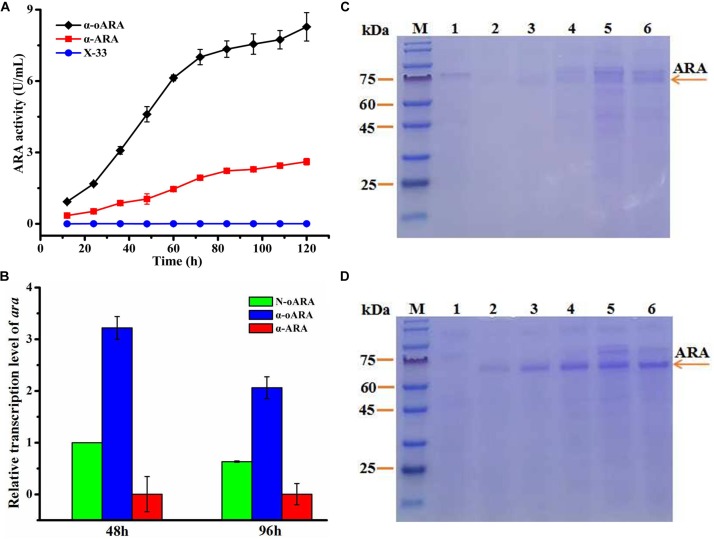
Effects of optimization of *ara* codons on target enzyme activity. **(A)** Extracellular enzyme activity of recombinant strains in flask culture. ARA activity of recombinant strain α-oARA (8.27 ± 0.60 U/mL) was 3.2-fold higher than that of α-ARA (2.61 ± 0.13 U/mL). **(B)** *ara* transcription level (determined by qPCR) in recombinant strains α-oARA and α-ARA. Optimization of *ara* codons (α-oARA) increased target gene transcription at both 48 and 96 h. **(C,D)** SDS–PAGE of ARA comparison of recombinant strains α-ARA **(C)** and α-oARA **(D)**. M, marker; 1, control (X-33); 2, 24 h; 3, 48 h; 4, 72 h; 5, 96 h; 6, 120 h.

Effects of optimized *ara* codons were investigated to enhance secretory expression of ARA in *P. pastoris*. Two expression plasmids were constructed: pPICZα-*ara* having one original *ara* codon, and pPICZα-*oara* having optimized *ara* codons. The transformed recombinant plasmids into *P. pastoris* (X-33), termed α-ARA and α-oARA, and confirmed by PCR and DNA sequencing (Supplementary Figure 1). Positive transformants and control strain without target gene (X-33) were cultured on agar plates of yeast extract YPD containing Zeocin (100 μg/mL), and further regulated by 1% methanol-induced AOX1 promoter. Following 96 h culture in BMMY medium, ARA activity of recombinant strain α-oARA (8.27 ± 0.60 U/mL) was 3.2-fold higher than that of α-ARA (2.61 ± 0.13 U/mL) (Figure 2A). SDS–PAGE analyses indicated similar molecular weights (60–75 kDa range) for ARA of recombinant strains α-oARA and α-ARA (Figures 2C,D).

Upregulation of target gene expression of optimized *ara* codons was confirmed by qPCR (Figure 2B). Therefore, selected recombinant strain α-oARA for further investigation.

### Propeptide Addition of Target Gene

**FIGURE 3:**
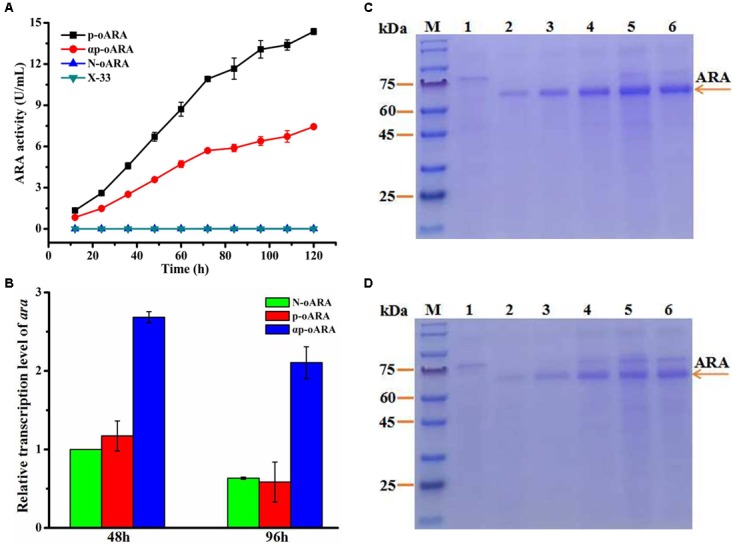
Comparison of ARA variants with different propeptides. **(A)** ARA activity of variants in flask culture. Extracellular enzyme activity of recombinant strain p-oARA (14.37 ± 0.22 U/mL) was 1.93-fold higher than that of αp-oARA (7.44 ± 0.12 U/mL). **(B)** Transcription level of *ara* (determined by qPCR) in recombinant strains p-oARA and αp-oARA. Addition of propeptide of target gene (p-oARA) reduced *ara* gene transcription at both 48 and 96 h. **(C,D)** SDS–PAGE of ARA comparison of recombinant strains p-oARA **(C)** and αp-oARA **(D)**. M, marker; 1, control (X-33); 2, 24 h; 3, 48 h; 4, 72 h; 5, 96 h; 6, 120 h.

The recombinant plasmids pPICZp-*oara* (with propeptide sequence but no α-factor signal peptide), pPICZαp-*oara* (with α-factor signal peptide and propeptide), and pPICZ-*oara* (without any peptide) were successfully constructed and transformed into *P. pastoris* (X-33) designated as p-oARA, αp-oARA and N-oARA, respectively (Supplementary Figure 1). Positive transformants and control strain without target gene (X-33) were cultured on YPD agar plates. ARA activity of recombinant strain p-oARA (14.37 ± 0.22 U/mL) was 1.93-fold higher than that of parental strain αp-oARA (7.44 ± 0.12 U/mL) (Figure 3A), while that of recombinant strain N-omARA was negligible (0.01 ± 0.001 U/mL). Target proteins were analyzed by SDS–PAGE, with X-33 as control (Figures 3C,D). ARA of recombinant strains p-oARA and αp-oARA each displayed a single, broad band with molecular weight range 60–75 kDa.

Transcription level of the three strains was analyzed by qPCR. The transcription level of *ara* (αp-oARA) was 2.29-fold higher than that of recombinant strain p-oARA (Figure 3B).

### Deglycosylation Analysis and *N*-terminal Amino Acid Sequencing

**FIGURE 4:**
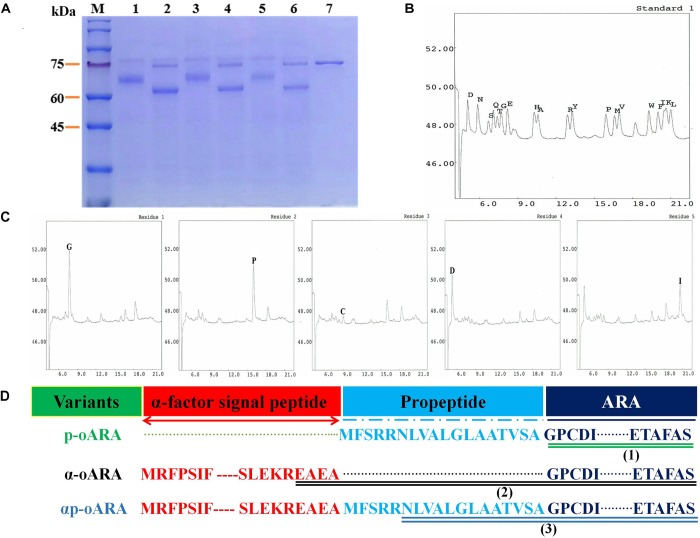
Deglycosylation analysis and N-terminal amino acid sequencing. **(A)** SDS–PAGE analysis of recombinant ARAs produced by the three recombinant strains following deglycosylation. M, marker; 1, p-oARA; 2, deglycosylated p-oARA; 3, α-oARA; 4, deglycosylated α-oARA; 5, αp-oARA; 6, deglycosylated αp-oARA; 7, endo-H. **(B,C)** N-terminal five amino acids of p-oARA culture supernatant. **(D)** Schematic illustration of variants fused with different propeptides. (1) mature ARA of recombinant strain p-oARA; (2) mature ARA of recombinant strain α-oARA; (3) mature ARA of recombinant strain αp-oARA.

To estimate the effect of glycosylation on enzyme activity, we performed ARAs deglycosylation of the three recombinant strains following endo-H treatment. After deglycosylation, ARA activity was reduced by 42.89% for recombinant strain p-oARA, by 73.14% for α-oARA, and by 60.93% for αp-oARA. Bioinformatics analysis indicated that ARAs containing different signal peptides and all exhibits three putative O-glycosylation sites (located at 44, 54, and 337) and two N-glycosylation sites (Asn 65 and Asn 184). More specifically, SDS–PAGE displayed difference in protein size after deglycosylation assay (Figure 4A, Lanes 2, 4 and 6), indicated the glycosylation effect on enzyme activity.

Results of N-terminal sequencing showed that secreted active ARA of recombinant strain p-oARA had theoretical initial five amino acids (GPCDI) (Figures 4B,C) comparable to the mature sequences of α-oARA (EAEAG) and αp-oARA (NLVAL) (Figure 4D and Supplementary Figures 2A,B).

### Expression of Recombinant ARA in the Fed-Batch 7.5-L Fermentor

**FIGURE 5:**
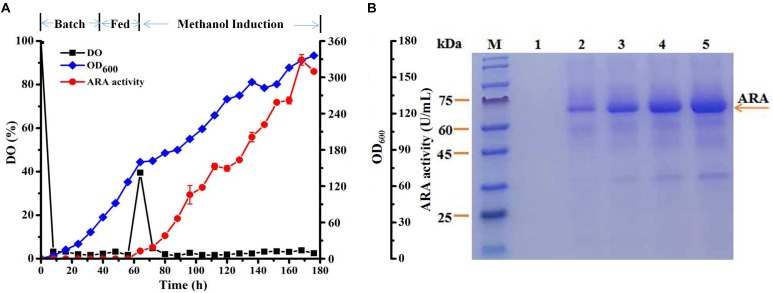
Expression of recombinant ARAs in fed-batch 7.5-L fermentor. **(A)** Time course profiles of recombinant strain p-oARA cultured in 7.5-L fermentor. ARA activity (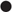), OD_600_ (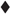), DO (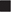). **(B)** SDS–PAGE analysis. M, marker; 1, control (X-33); 2, 24 h; 3, 48 h; 4, 72 h; 5, 96 h.

Secretory expression of ARA by three recombinant strains p-oARA, α-oARA and αp-oARA was significantly enhanced in shaking flask fermentation. We also performed fed-batch fermentation of the three strains in a 7.5-L fermentor. ARA activity of recombinant strain p-oARA at 168 h was 164.47 ± 4.40 U/mL (Figure 5A), which was 2.53-fold higher than α-oARA (64.85 ± 1.52 U/mL) and 2.34-fold higher than that of αp-oARA (70.30 ± 4.76 U/mL) (Supplementary Figures 3A,C). The extracellular culture supernatant of three recombinant strains p-oARA, α-oARA and αp-oARA were analyzed by SDS–PAGE as shown in Figure 5B and Supplementary Figures 3B,D. ARA specific activity of recombinant strain p-oARA (479.50 ± 12.83 U/mg) was significantly higher than other two strains α-oARA (329.19 ± 7.72 U/mg) and αp-oARA (371.96 ± 25.18 U/mg).

### Enzyme Properties

**FIGURE 6:**
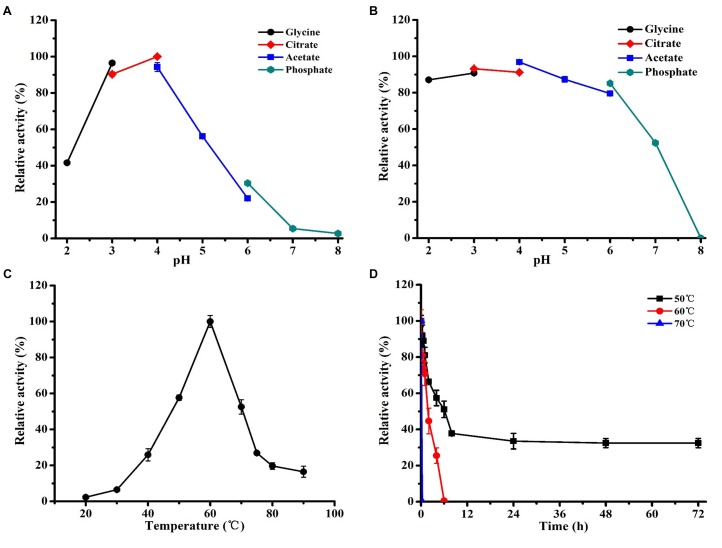
Effects of temperature and pH on ARA activity of recombinant strain p-oARA. **(A)** Optimal pH values for 50 mM solutions of glycine-HCl (pH 2-3), sodium citrate (pH 3-4), sodium acetate (pH 4-6), and sodium phosphate (pH 6-8). **(B)** pH stability was determined in various buffers as in (A) following incubation for 1 h at 4°C, and residual activity was measured. **(C)** Optimal temperature determined in sodium citrate buffer (pH 4.0). **(D)** Enzyme stability in sodium citrate buffer (pH 4.0), at three temperatures as indicated. All determinations were performed in triplicate.

Enzyme properties of recombinant strain p-oARA culture supernatant were evaluated using the substrate of pNP-α-L-Araf. ARA was active over a wide pH range (2.0 to 8.0), with optimal pH 4.0 (Figure 6A). After 1 h incubation at room temperature, ARA activity was still near 80% of maximal value in pH range 2.0–6.0 (Figure 6B).

In temperature-dependent ARA activity of recombinant strain p-oARA assay at pH 4.0, activity was highest at 60°C (Figure 6C). In thermostability assay, ARA showed maximal stability at 50°C for 72 h, and was rapidly inactivated (with half-life 2 h) above 60°C (Figure 6D).

When effects of chemical reagents and various metal ions were examined (Supplementary Table 2), ARA activity of recombinant strain p-oARA was significantly improved in the presence of Fe^2+^ and Tween-20, inhibited in the presence of Fe^3+^ and SDS, and unaffected by EDTA.

α-L-arabinofuranosidase activity of recombinant strain p-oARA showed most active against pNP-α-L-Araf (164.47 ± 4.40 U/mL) and moderate activity on pNPX (0.68 U/mL). No activity was detected toward RBB-xylan, pNPG, pNPC and CMC-Na (data not shown).

Kinetics parameters of ARA were evaluated for the hydrolysis of the pNP-α-L-Araf substrate using various concentrations (0.5 to 5.0 mM). ARA activity of recombinant strain p-oARA exhibited the *V*_max_ and *K*_m_ of 747.55 μmol/min/mg and 5.36 mmol/L, which was significantly higher enzymatic efficiency than that of strains α-oARA and αp-oARA (Supplementary Table 3).

### Saccharification of Pretreated Corn Stover

**FIGURE 7:**
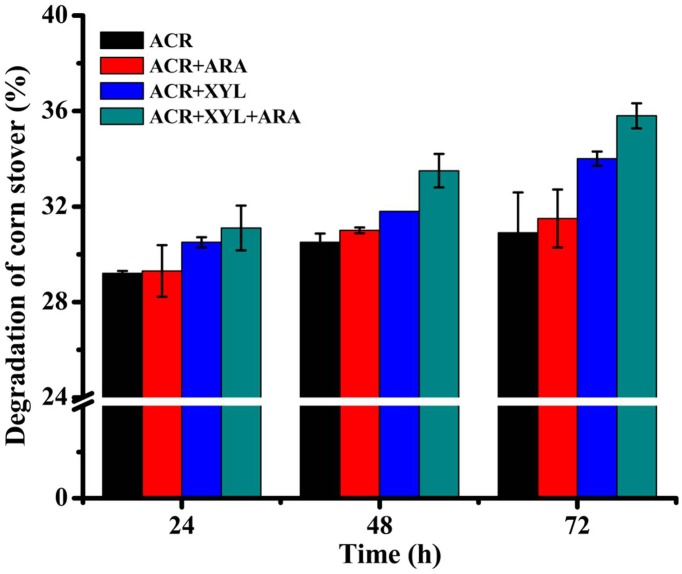
Degradation of corn stover (%) in enzymatic hydrolysis assays performed for 24, 48, and 72 h at 50°C. Addition of ARA (dosage 16.8 U per g biomass) and XYL (dosage 80 U per g biomass) significantly increased saccharification in synergistic fashion. Enzymatic hydrolysis yield was 15.9% higher for ARA + XYL addition than for ACR mixture.

α-L-arabinofuranosidase of recombinant strain p-oARA displayed synergistic action with a hemicellulolytic enzyme (XYL) in degradation of pretreated corn stover (Figure 7). In the reaction mixture, ACR (dosage 1.18 FPU per g biomass) degraded 30.9% of corn stover during 72 h incubation at 50°C, pH 5.0. Addition of ARA (dosage 16.8 U per g biomass) and XYL (dosage 80 U per g biomass) significantly increased saccharification in a synergistic fashion, these enzymes degraded 31.5% and 34% of corn stover, respectively. Degree of synergism for ARA + XYL was determined as 32.4%. Enzymatic hydrolysis yield, also known as reducing sugar conversion, for ARA + XYL addition, was 15.9% higher than that of ACR.

## Discussion

Arabinofuranosidases (ARAs) are produced by a variety of lignocellulolytic fungi and bacteria, including *A. nidulans, A. niger, T. reesei* Rut-C30 and *Thermotoga thermarum* (Gielkens et al., 1999; de Souza et al., 2011; Marx et al., 2013; Shi et al., 2013). Members of the genus *Aspergillus* are an important source of CAZymes having high protein secretion capability. Numerous ARAs belonging to various GH families have been isolated from *Aspergillus* species; These ARAs display varied enzyme properties and PTM capabilities (Benoit et al., 2015). ARAs belonging to the GH54 family are particularly useful for plant biomass degradation. A novel recombinant ARA from *A. niger* ND-1 produced by *P. pastoris* expression system in the present study was assigned to the GH54 family according to the CAZy databases.

In recent studies, high yields of recombinant ARAs have been achieved based on engineering and enhanced expression of ARAs using *P. pastoris* expression system (Amore et al., 2012; Yang et al., 2015b). Different microorganisms have different codon usage, and replacing with preferred codons of *P. pastoris* is an effective strategy for increasing protein expression levels (Zhao et al., 2011). Likewise, codon optimization can greatly alter expression levels and properties of enzymes (Li et al., 2012). Optimization of *T. reesei* endoglucanase gene led to enzyme activity 24% higher than that associated with the native gene (Bayram et al., 2011). This strategy has not been previously applied for enhancing secretory expression of ARAs in *P. pastoris*. For this purpose, we successfully performed optimization of *ara* codons, based on the construction of codon-optimized expression plasmid pPICZα-o*ara*. ARA activity of recombinant strain α-oARA was 3.2-fold higher than that of original expression strain α-ARA. These findings are consistent with a recent report that expression and secretion of target heterologous protein is enhanced by optimization of *ara* codons (Yang and Zhang, 2018).

Attempts to develop yeast strains with improved yield typically face strain- and product-specific challenges involving recruitment of natural propeptides, gene dosages, and oxygen demand (Nocon et al., 2014). Strategies in which genetic and process engineering approaches are combined have been used to overcome such challenges (Wang et al., 2010). Signal peptide sequences have been evaluated to improve protein expression levels in *P. pastoris*, with particular focus on secretion efficiency and optimization of α-factor signal peptide codons (Huang et al., 2014). Trypsin production in *P. pastoris* was increased from 6.87 ± 0.11 U/mL to 12.64 ± 0.51 U/mL by recruitment of natural propeptides for SGT self-activation (Zhang et al., 2018). ARA activity of recombinant strain p-oARA (14.37 ± 0.22 U/mL) was 1.93-fold higher than parental strain αp-oARA (7.44 ± 0.12 U/mL). p-oARA may have advantages over αp-oARA and α-oARA strains in regarding protein modifications, transport pathways, stability of mRNA structure, and unfolded protein response related genes, in analogy to previous findings (Faure et al., 2016).

In *P. pastoris*, glycosylation is the major PTM of secretory proteins and has significant effect on the properties of various enzymes. We treated recombinant ARA with endo-H to assess effects of glycosylation on enzyme activity. The recombinant protein was glycosylated, with consequent significant effects on enzyme activity, similarly to previous findings for other fungal hemicellulases, e.g., endoxylanases and β-xylosidases (Birijlall et al., 2011; Wongwisansri et al., 2013).

Protein secretion in yeast follows a pathway from ER to Golgi to extracellular space. An essential step in this process is site-specific cleavage of the signal peptide from preprotein. Differences in amino acid residues may greatly impact cutting efficiency and consequently the final amount of secretory proteins (Mohamed et al., 1995). N-terminal sequencing results in this study showed that secreted ARA of p-oARA shared theoretical initial five amino acids (GPCDI) relative to mature sequences of α-oARA (EAEAG) and αp-oARA (NLVAL). We tried to insert propeptide after α-factor signal peptide to enhance the ARA expression level, however, no effect was observed on the results. Therefore, the secreted protein of the strain αp-oARA has a messed up sequence at the N-terminal is completely expected.

In 7.5-L fermentor, extracellular protein concentration for recombinant strain p-oARA (0.343 mg/mL) was almost twice as high as for αp-oARA (0.189 mg/mL) or α-oARA (0.197 mg/mL). qPCR analysis revealed target gene transcription levels for recombinant strains α-oARA and αp-oARA that were, respectively, 2.73- and 2.29-fold higher than p-oARA. More specifically, the recombinant strains (p-oARA, αp-oARA and α-oARA) were verified as a single copy integrated into *P. pastoris* genome by PCR. Previous studies have shown that N-terminal amino acid for most natural proteins is translated at a low rate, allowing sufficient time for nascent amino acid chains to fold correctly into specific domains (Shah et al., 2013). This phenomenon maybe account for the fact that p-oARA had lower translation rate but higher production of the extracellular target protein (Goodman et al., 2013). Our findings, taken together, indicate that introduction of propeptide is a feasible and effective technique for enhancing ARA activity.

Factors that affect enzyme fed-batch fermentation performance include medium composition, feeding strategy, carbon source, temperature and pH (Garcia-Ortega et al., 2013). In our fed-batch fermentation experiments, consistent with findings from flask culture, ARA activity for recombinant strain p-oARA increased steadily and reached a value of 164.47 ± 4.40 U/mL at 168 h, 2.53-fold higher than that of α-oARA (64.85 ± 1.52 U/mL) and 2.34-fold higher than that of αp-oARA (70.30 ± 4.76 U/mL). However, solely based on the hydrolysis of pNPX and pNP-α-L-Araf, the *Hypocrea koningii* GH 54 enzyme known as a bifunctional arabinofuranosidase/xylosidase (Wan et al., 2007). Furthermore, the comparision of debranched and branched arabinan hydrolysis, the activity always found non-existent or lower on the debranched form (Sakamoto and Kawasaki, 2003). In particular, the high specific enzyme activity using pNP-α-L-Araf as substrate was only 60 U/mg from *T. reesei* (Margolles-Clark et al., 1996). In this sudy, because heterologous protein expression in the culture supernatant was homogeneous, we used supernatant directly (no further purification) for biochemical characterization of enzyme activity. ARA of recombinant strain p-oARA showed optimal pNP-α-L-Araf hydrolysis performance at 60°C, pH 4.0, with specific activity 479.50 ± 12.83 U/mg and moderate activity on pNPX (1.98 U/mg) in the supernatant. These findings are comparable to those for other ARAs that show optimal activity under acidic and mesophilic conditions (Flipphi et al., 1993a,b; Sánchez-Torres et al., 1996).

Synergistic action has been demonstrated for many enzymes (cellulolytic and hemicellulolytic) from various fungi and bacteria (Peng et al., 2015; Valadares et al., 2016), and synergistic action of ARA with other hemicellulases has been observed in degradation of substrates (Birijlall et al., 2011; Yang et al., 2015a). We experimented with combinations of enzymes XYL and ARA to improve the efficiency of corn stover degradation. Hydrolysis of arabinoxylans by ARA enhanced the ability of XYL to degrade corn stover. Consequently, Enzymatic hydrolysis yield was 15.9% higher for ARA + XYL addition than for ACR mixture. Similar results have been obtained for ARAs from *Humicola insolens* Y1 (Birijlall et al., 2011) and *Paenibacillus* sp. E18 (Sha et al., 2013). One possible explanation is that removal of side chains by ARA increases accessibility of the xylan backbone to xylanase, thereby enhancing degradation efficiency (Jørgensen et al., 2010). Improved hydrolysis efficiency and removal of hemicellulose debris may exert a synergistic effect by increasing accessibility of cellulose fibers to cellulases and reducing product inhibition, thus decreasing required enzyme dosages.

## Conclusion

In this study, a recombinant *P*. *pastoris* strain was developed, through *ara* codon optimization and N-terminal propeptide engineering, for improved secretory production of ARA from *A*. *niger* ND-1. Engineered strain p-oARA displayed high specific activity and yield 5.5-fold higher than that of parental strain α-ARA. The highest specific activity and production values for ARA in *P*. *pastoris* were firstly reported so far. A native propeptide (MFSRRNLVALGLAATVSA) enhancing extracellular protein concentration for ARA and enzymatic efficiency was superior. Degree of synergism for ARA and xylanase was remarkable on corn stover degradation, indicating great potential of ARA for biofuel production.

## Author Contributions

FZ and JL designed the study and worked on the molecular genetic studies. FZ carried out the experiments of enzymatic properties. FZ, TM, and JL helped in fermentation. AB and FZ prepared the manuscript. WJ supervised the present study.

## Conflict of Interest Statement

The authors declare that the research was conducted in the absence of any commercial or financial relationships that could be construed as a potential conflict of interest.

## Footnotes

1 http://www.cazy.org

2 http://www.ncbi.nlm.nih.gov/BLAST/

3 http://www.cbs.dtu.dk/services/NetNGlyc/

4 http://www.cbs.dtu.dk/services/SignalP/

5 http://www.jcat.de/

## Abbreviations

ACR: Celluclast 1.5L
ARAs: α-L-arabinofuranosidases
CAZymes: carbohydrate-active enzymes
CMC-Na: sodium carboxymethyl cellulose
DNS: dinitrosalicylic acid
DO: dissolved oxygen
GH: glycoside hydrolase
pNPG: p-nitrophenyl-β-D-galactopyranoside
pNP-α-L-Araf: p-nitrophenyl-α-L-arabinofuranoside
pNPC: p-nitrophenyl-β-D-cellobioside
pNPX: P-nitrophenyl (pNP) -β-D-xylopyranoside
PTMs: post-translational modifications
qPCR: quantitative real-time RT-PCR
RBB-xylan: remazol brilliant blue-xylan
XYL: endo-1,4-β-xylanase
