# Supplementary material for: Insight to Improve α-L-Arabinofuranosidase Productivity in Pichia pastoris and Its Application on Corn Stover Degradation
Source: Front Microbiol. 2018 Dec 14;9:3016. doi: 10.3389/fmicb.2018.03016 (PMC6315152; doi:10.3389/fmicb.2018.03016)
Supplement: Supplementary file 2 [file Data_Sheet_2.PDF]

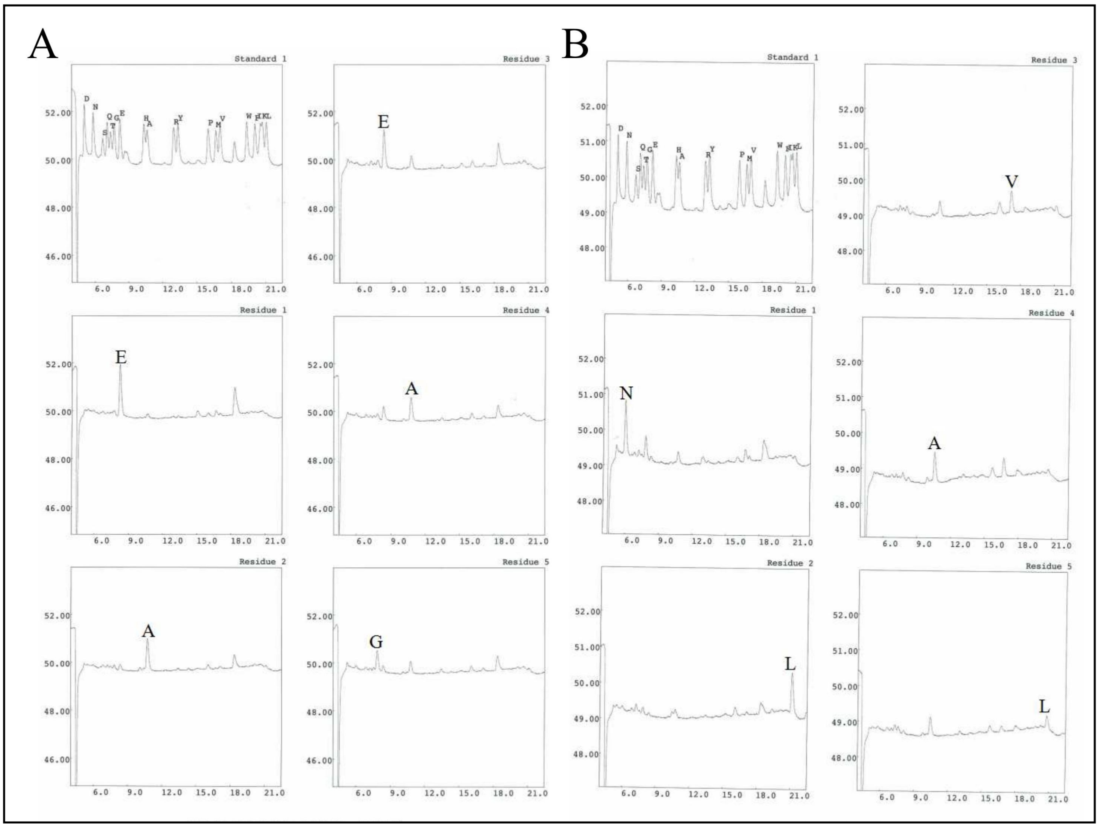

Supplementary Figure 2. N-terminal five amino acids of recombinant strains α-oARA (**A**) and αp-oARA (**B**) in culture supernatant. The mature ARA sequences of recombinant strains α-oARA and αp-oARA were origin from EAEAG and NLVAL, respectively.
