# Supplementary material for: Insight to Improve α-L-Arabinofuranosidase Productivity in Pichia pastoris and Its Application on Corn Stover Degradation
Source: Front Microbiol. 2018 Dec 14;9:3016. doi: 10.3389/fmicb.2018.03016 (PMC6315152; doi:10.3389/fmicb.2018.03016)
Supplement: Supplementary file 3 [file Data_Sheet_3.PDF]

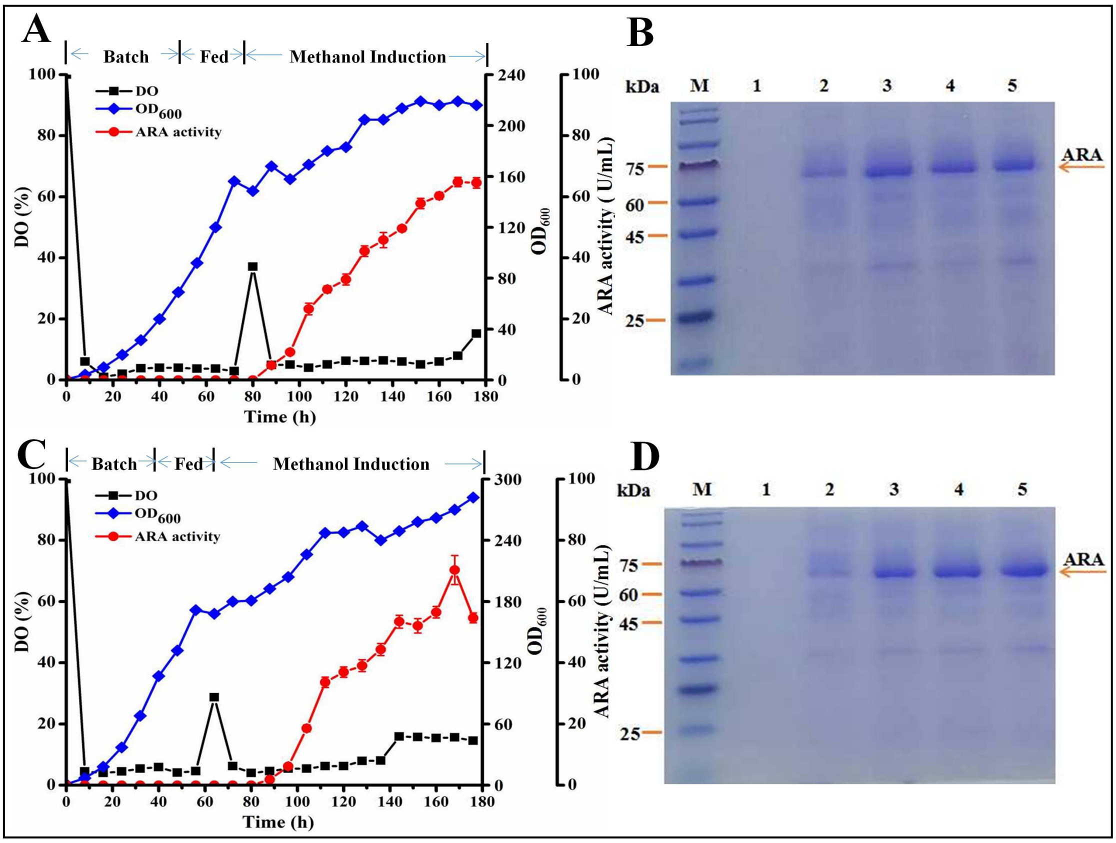

Supplementary Figure 3. Expression of recombinant ARAs in fed-batch 7.5-L fermentor. (**A, C**) Time course profiles of recombinant strains α-oARA (**A**) and αp-oARA (**C**) cultured in 7.5-L fermentor. ARA activity (●), OD600 (◆), DO (■). (**B, D**) SDS-PAGE analysis. M, marker; 1, control (X-33); 2, 24 h; 3, 48 h; 4, 72 h; 5, 96 h.
