# Supplementary material for: Insight to Improve α-L-Arabinofuranosidase Productivity in Pichia pastoris and Its Application on Corn Stover Degradation
Source: Front Microbiol. 2018 Dec 14;9:3016. doi: 10.3389/fmicb.2018.03016 (PMC6315152; doi:10.3389/fmicb.2018.03016)
Supplement: Supplementary file 4 [file Data_Sheet_4.PDF]

Supplementary Table 1. Oligonucleotide primers used in this study.

Primers	Sequence (5´- 3´)
ARA-F	GAATTCCCCTGTGACATCTACGAACG
ARA-R	TCTAGACCCGAAGCAAACGCCGTCTC
pARA-F	GAATTCATGTTCTCCCGCCGAAACC
pARA-R	TCTAGACCCGAAGCAAACGCCGTCTC
AOX-F	GACTGGTTCCAATTGACAAGC
AOX-R	GCAAATGGCATTCTGACATCC
pUC-F	AGCAGATTACGCGCAGAAAAAAAG
ara internal reverse primer-R	CTTACTCCGCTGTA ATATGTACTC
**ARA Variants**	
pPICZp-*oara*-F	AACTAATTATTCGAAACGATGTTCTCCAGAAGGAAT
pPICZp-*oara*-R	ATTCCTTCTG GAGAACATCGTTTCGAATAATTAGTT
pPICZα-*oara*-F	AAAAGAGAGGCTGAAGCTGGTCCTTGCGATATTTAT
pPICZα-*oara*-R	ATAAATATCGCAAGGACCAGCTTCAGCCTCTCTTTT
pPICZ-*oara*-F	AACTAATTATTCGAAACGGGTCCTTGCGATATTTAT
pPICZ-*oara*-R	ATAAATATCGCAAGGACCCGTTTCGAATAATTAGTT
**Real-time PCR**	
F_{rARA}	GTGTCTTTATGTCTCCCGGTAC
R_{rARA}	CTCCATAACCCCAAGCAGTAG
F_{GAPDH}	TACGTCATTGAGTCCACCGGT
R_{GAPDH}	TGGTAGTACAAGAAGCATTGGAG
