# Supplementary material for: Insight to Improve α-L-Arabinofuranosidase Productivity in Pichia pastoris and Its Application on Corn Stover Degradation
Source: Front Microbiol. 2018 Dec 14;9:3016. doi: 10.3389/fmicb.2018.03016 (PMC6315152; doi:10.3389/fmicb.2018.03016)
Supplement: Supplementary file 5 [file Data_Sheet_5.PDF]

Supplementary Table 2. Effects of metal ions (5 mM) and chemical reagents on ARA activity of recombinant strain p-oARA.

Chemicals	Relative activity (%)	Chemicals	Relative activity (%)
Control	100.00 ± 1.25	Mg^{2+}	104.74 ± 0.55
Ni^{2+}	97.73 ± 0.44	EDTA	95.32 ± 0.56
Co^{2+}	101.98 ± 0.22	Cu^{2+}	94.40 ± 0.11
Al^{3+}	100.07 ± 0.55	Ca^{2+}	112.18 ± 1.1
Na^{+}	96.50 ± 0.74	Fe^{3+}	86.50 ± 0.86
Mn^{2+}	103.44 ± 1.8	SDS (0.1%)	79.79 ± 0.09
Zn^{2+}	103.25 ± 0.44	Tween-20 (0.05%)	113.95 ± 0.11
Fe^{2+}	137.03 ± 1.43		
