# Supplementary material for: Insight to Improve α-L-Arabinofuranosidase Productivity in Pichia pastoris and Its Application on Corn Stover Degradation
Source: Front Microbiol. 2018 Dec 14;9:3016. doi: 10.3389/fmicb.2018.03016 (PMC6315152; doi:10.3389/fmicb.2018.03016)
Supplement: Supplementary file 6 [file Data_Sheet_6.PDF]

Supplementary Table 3. Enzyme kinetics parameters of ARA expressed in three variants.

**Variants**	**K_m (mmol/L)**	**V_{max} (μmol/min/mg)**
p-oARA	5.36	747.55
α-oARA	6.07	685.99
αp-oARA	6.54	745.24
