# Supplementary material for: Insight to Improve α-L-Arabinofuranosidase Productivity in Pichia pastoris and Its Application on Corn Stover Degradation
Source: Front Microbiol. 2018 Dec 14;9:3016. doi: 10.3389/fmicb.2018.03016 (PMC6315152; doi:10.3389/fmicb.2018.03016)
Supplement: Supplementary file 1 [file Data_Sheet_1.PDF]

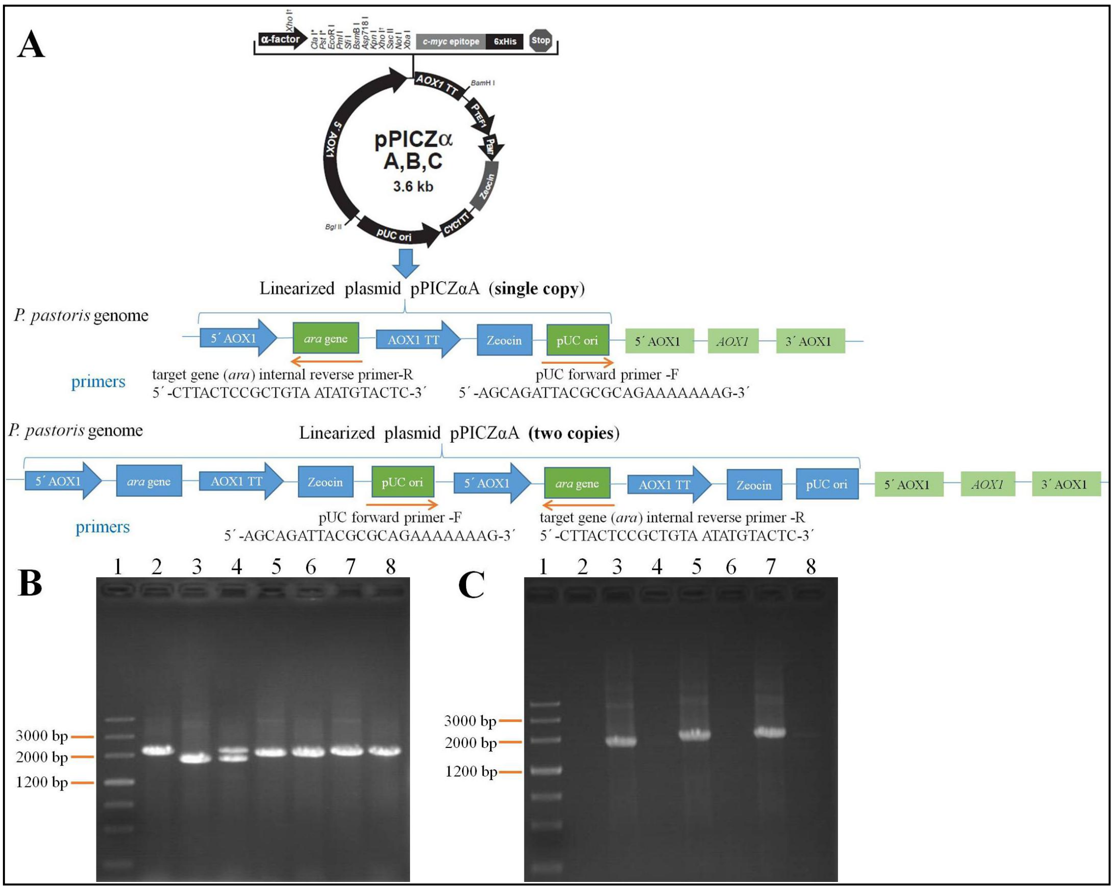

Supplementary Figure 1. The confirmation of the recombinants by PCR. (**A**) Schematic diagram of single exchange homologous recombination of *P. pastoris*. (**B**) The identification of positive transformants and control strains without target gene (X-33) using PCR primer AOX-F/ AOX-R and sequencing. **1**, marker; **2**, control strain X-33; **3**, recombinant plasmid pPICZp-*oara*; **4**, recombinat strain p-oARA; **5**, recombinant plasmid pPICZα-*oara*; **6**, recombinat strain α-oARA; **7**, recombinant plasmid pPICZαp-*oara*; **8**, recombinat strain αp-oARA. (**C**) The verification of single copy *P. pastoris* recombinants by PCR with primer pair (pUC-F: 5´-AGCAGATTACGCGCAGAAAAAAAG-3´; target gene (*ara*) internal reverse primers-R: 5´-CTTACTCCGCTGTA ATATGTACTC-3´). **1**, marker; **2**, control strain X-33; **3**, recombinant plasmid pPICZp-*oara*; **4**, recombinat strain p-oARA; **5**, recombinant plasmid pPICZα-*oara*; **6**, recombinat strain α-oARA; **7**, recombinant plasmid pPICZαp-*oara*; **8**, recombinat strain αp-oARA.
